# Key questions on the long term renal effects of lithium: a review of pertinent data

**DOI:** 10.1186/s40345-023-00316-5

**Published:** 2023-11-16

**Authors:** Michael Gitlin, Michael Bauer

**Affiliations:** 1 Department of Psychiatry and Biobehavioral Sciences, The Semel Institute for Neuroscience and Human Behavior, University of California Los Angeles (UCLA), Los Angeles, CA USA; 2 Department of Psychiatry and Psychotherapy, Faculty of Medicine, University Hospital Carl Gustav Carus, Technische Universität Dresden, Fetscherstr. 74, 01307 Dresden, Germany

**Keywords:** Bipolar disorder, lithium, Kidney function, Maintenance treatment

## Abstract

For over half a century, it has been widely known that lithium is the most efficacious maintenance treatment for bipolar disorder. Despite thorough research on the long-term effects of lithium on renal function, a number of important questions relevant to clinical practice remain. The risk of polyuria, reflecting renal tubular dysfunction, is seen in a substantial proportion of patients treated with long term lithium therapy. The duration of lithium may be the most important risk factor for lithium-induced polyuria. Most, but not all, studies find that lithium is associated with higher rates of chronic kidney disease compared to either age matched controls or patients treated with other mood stabilizers. Age, duration of lithium therapy and medical disorders such as hypertension and diabetes mellitus are risk factors for chronic kidney disease in lithium-treated patients. The relationship between polyuria and chronic kidney disease is inconsistent but poorly studied. Although not all studies agree, it is likely that lithium may increase the risk for end stage renal disease but in a very small proportion of treated patients. Patients whose renal function is relatively preserved will show either no progression or improvement of renal function after lithium discontinuation. In contrast, patients with more renal damage frequently show continued deterioration of renal function even after lithium discontinuation. Optimal management of lithium treatment requires obtaining a baseline measure of renal function (typically estimated glomerular filtration rate [eGFR]) and regular monitoring of eGFR during treatment. Should the eGFR fall rapidly or below 60 ml/minute, patients should consider a consultation with a nephrologist. A decision as to whether lithium should be discontinued due to progressive renal insufficiency should be made using a risk/benefit analysis that takes into account other potential etiologies of renal dysfunction, current renal function, and the efficacy of lithium in that individual patient.

## Introduction

Despite the fact that lithium is consistently ranked as the first choice medication for the long-term treatment of bipolar disorders in international guidelines lithium prescriptions are declining in many countries (Malhi et al. 2023). Alterations of renal function from lithium have been well described for a very long time. Close to a century before Cade’s first seminal report on the efficacy of lithium in treating acute mania (Cade 1949), the diuretic effect of lithium (clinically manifested as polyuria) was noted in a number of reports in the 1860s (summarized in Johnson, 1980), suggesting its use as a clinical diuretic in disorders such as gout. In 1977, ‘the kidney scare” from lithium emerged from preliminary biopsy studies of selected lithium- treated patients which showed clear evidence of structural renal damage, primarily in the form of interstitial nephritis (with scarring of the interstitium, tubular destruction and relative preservation of glomeruli [Hestbech et al. 1977]). As noted by Schou and Vestergaard three years later, clinicians were appropriately concerned about these findings, asking “are we buying the mental health of lithium-treated manic-depressive patients at the expense of their kidney function and survival? Should we perhaps stop using lithium?” (Schou and Vestergaard, 1980). Their answer was, not surprisingly “no”. After acknowledging the data available at that time on both polyuria and potential morphological kidney changes during lithium treatment, they appropriately recommended that these effects do not justify radical changes in the use of lithium. Surprisingly, they did not consider monitoring of renal function such as serum creatinine determinations mandatory.

Now, forty three years later, we have learned a great deal more about the renal effects of lithium and the potential risks of long term renal damage. Yet, the questions generated almost half a century ago are still relevant and will be addressed in this paper:

1. What is the risk of polyuria among lithium-treated patients?
2. What is the risk of mild to moderate renal damage from lithium as measured by the estimated glomerular filtration rate (eGFR)?
3. What is the relationship between polyuria/nephrogenic diabetes insipidus and the development of chronic kidney disease (CKD) among a lithium treated population?
4. What is the risk of end stage renal disease (ESRD), defined as an eGFR < 15 ml/min, in patients taking lithium?
5. What is the course of renal function in lithium treated patients who discontinue lithium due to CKD?

### Risk of polyuria among lithium-treated patients

Urinary frequency, i.e., polyuria, is the most common side effect of lithium. Estimates of polyuria vary with estimates up to 70% with a range of 20–87% (Goldberg and Ernst 2012; Azab et al. 2015). Nephrogenic diabetes insipidus (NDI), also known as arginine vasopressin resistance (AVP-R), defined as a 24 h urine volume of 3,000 ml/day or more is both measured less frequently and is less prevalent with estimates ranging between 3 and 17% (Damba et al. 2022). Thirst and polyuria are not always well correlated with formally defined NDI, especially in older patients (Rej et al., 2014a). Additionally, patients are often imprecise and even mistaken in their subjective sense of increased urination/polyuria. Although difficult, a formal 24 h urine collection is the definitive method for ascertaining the presence of polyuria. The overall mechanism by which lithium causes NDI is thought to be resistance to antidiuretic hormone (ADH), especially through its effects at the distal tubules and collecting ducts (Davis et al. 2018a; Lerma, 2023). More specifically, lithium accumulates in the principal cells of the collecting duct, reducing the phosphorylation of aquaporin 2 (AQP2), thereby reducing the reabsorption of free water and resulting in the excretion of a more dilute urine. (See Davis et al. 2018a for more details).

Factors that have been reported to increase the risk of lithium induced polyuria include: duration of lithium treatment; serum lithium level; and number of episodes of lithium intoxication. It is thought that the polyuria is initially functional (similar to what is seen with alcohol use) and reversible but becomes structural and less reversible with time. It has been estimated that the polyuria is usually reversible in the first 2–6 years of treatment (Schoot et al. 2020). Concomitant use of antipsychotics has been reported to increase the risk of lithium-induced polyuria.

The regimen by which lithium is prescribed may also correlate with polyuria. Both animal studies and studies in clinical populations suggest that lithium induced damage to the renal tubular systems is minimized by regimens that result in a very low daily trough lithium level (Gitlin 1999). Rats treated with lithium at a relatively constant concentration show reduced renal concentrating ability and greater tubular and interstitial scarring on biopsy specimens compared to rats treated with a regimen producing high peaks alternating with low trough lithium concentrations (Plenge et al. 1981). Clinically, low trough levels can best be achieved by once daily administration of non-sustained release lithium carbonate capsules. Thus, even though it is superficially paradoxical, steadier lithium levels across a 24 h period, which would be obtained by a regimen of twice or three times daily lithium administration using a sustained release preparation may result in greater tubular damage and therefore higher rates of polyuria/NDI compared to once daily lithium capsules. Some early studies, however, did not demonstrate improvement in polyuria when patients were switched to a once daily lithium regimen except in those who had been treated with lithium for five years or less (Kusalic et al., 1996), again suggesting the gradual transition from a functional to a structural, less reversible deficit. Therefore, for patients who have been treated for lithium on twice daily regimens for long time periods-e.g. ten years or more, switching to a once daily regimen may not be beneficial. Since CNS lithium levels vary far less throughout a 24 h period than does the serum level, the high peak/low trough pharmacokinetic curve does not interfere with lithium’s efficacy.

For patients with lithium induced polyuria which is symptomatically distressing and functionally impairing-e.g. sleep disruption due to recurrent nocturia, both amiloride (Batlle et al. 1985) and thiazide diuretics can be helpful. Amiloride inhibits the uptake of lithium in the principal cells of the epithelial sodium-channel while thiazides reduce distal delivery of tubular fluid resulting from increased proximal sodium and water uptake (Schoot et al. 2020). Thiazides increase serum lithium levels, thereby typically requiring a decrease in daily lithium dose to maintain constant levels.

### Risk of renal damage (CKD) as measured by eGFR

Although polyuria is distressing and may decrease quality of life-especially in patients with marked nocturia disrupting sleep- as long as patients respond to thirst and have access to sufficient liquids to stay hydrated, it is not a substantial health risk. However, with progressive scarring from the primarily interstitial disease associated with long term lithium use, glomerular function may begin to be compromised. Most but not all studies use CKD stage 3 defined as eGFR < 60 ml/minutes as the cutoff when dichotomizing renal function. Table 1 presents current classification of chronic kidney disease. Two important confounding factors are bipolar disorder itself and age. Bipolar disorder itself is associated with a decrease in average life span and specifically in rates of mild to moderate CKD as well as end stage renal disease (Kessing et al. 2015). Normal aging is also associated with the development of mild to moderate CKD-3. Normal age-related renal function deterioration has been estimated as decreasing eGFR of 0.4 ml/minute/year (Wetzel et al., 2007; Hill et al. 2016).

**Table 1:** Stages of Chronic Kidney Disease

Stage	GFR (mL/min/1.73^m2^)
1	≥ 90
2	60–89
3	30–59
4	15–23
5	< 15 **(dialysis or transplantation)**

A number of recent reviews have surveyed the field, addressing the rate of CKD stage 3 in lithium treated patients and compared them to either age matched controls or to patients treated with other mood stabilizing agents (Lerma 2023; Schoretsanitis et al., 2021; Damba et al. 2022; Schoot et al. 2020). A recent estimate of the rate of CKD stage 3 in lithium treated patients was 26% (Schoretsanitis et al., 2021). A review of 15 papers found that all 15 studies found a positive association between CKD stage 3 and lithium with cumulative incidences ranging between 2 and 40% (Damba et al. 2022). Two studies each estimated the rate of CKD-3 was 30% higher in lithium treated patients compared to a control population (Tondo et al. 2017; Van Alphen et al. 2021). Another review estimated that the risk of CKD Stages 3–5 was five fold higher in patients on long term lithium treatment (Schoretsanitis et al., 2021). Most, but not all studies (Kessing et al. 2015; Bosi et al. 2023) found that lithium was associated with higher rates of CKD 3 compared to other mood stabilizing agents.

Other than age (which is a risk factor for CKD 3 for all populations), the most consistent risk factor for lithium-induced renal disease is length of exposure to lithium (Hojlund et al. 2022). Despite occasional reports of lithium induced CKD after relatively shorter exposures-i.e., years, not decades (Damba et al. 2022; Gitlin 2023), most patients with CKD have been on lithium for twenty years or more. One recent study demonstrated that the average annual decline in eGFR in lithium treated patients differed from a control population only after ten years of lithium exposure (Fransson et al. 2022). Other risk factors found in most but not all studies are episodes of lithium toxicity, higher lithium levels, the use of other psychotropic medications and comorbid medical disorders associated with renal damage such as diabetes mellitus and hypertension (Damba et al. 2022; Hojlund et al. 2022). Other potential risk factors recently identified are female sex (which increases the risk for CKD generally) and a history of migraines (Hayes et al. 2021).

Although virtually all Practice Guidelines recommend regular (between every three months to every year, depending on the guidelines) monitoring of renal function, in clinical practice, monitoring of lithium levels and renal function in lithium treated patients may be far less frequent. As one example, in a French study, during an eight year period, of 1179 lithium treated patients, 41% had no creatinine measurement (Bassilios et al. 2008). The overall recommendations for lower lithium levels-0.6-0.8 mEq/ml vs. 0.8-1.0mEq/ml- should also be the goal for most patients. Even one lithium level > 1.0 mEq/L has been reported to be associated with a lower eGFR for three months (Kirkham et al. 2014). Whether this reflects a transient or a longer term effect on renal function is unknown.

### Relationship between polyuria/ nephrogenic Diabetes insipidus and the development of chronic Kidney Disease (CKD)

Since the mechanism by which lithium causes both polyuria/NDI and CKD are presumed to be the same-interstitial nephritis- it may equally be assumed that the presence of polyuria/NDI would be a strong risk factor for the development of CKD. Surprisingly, this has been rather infrequently studied. One study found a correlation between low urine specific gravity (a marker for NDI) with long term renal decline as measured by a lower eGFR (Sajadi et al., 2016). Neither of the most recent comprehensive reviews evaluated polyuria as a predictor of CKD in lithium treated patients. (Schoretsanitis et al., 2021; Damba et al. 2022). An earlier review simply stated that “polyuria only weakly predicts increasing creatinine or reduced kidney function” (Azab et al. 2015). Many clinicians have anecdotally seen patients who have substantial and distressing polyuria but whose eGFR is normal (> 80 ml/min). Of note, some bipolar patients may have psychogenic polydipsia in which the polydipsia is primary and the polyuria is secondary, whereas with lithium the polyuria is primary. Whether this reflects the relative preservation of glomeruli compared to tubular damage consistent with interstitial nephritis or is the consequence of additional factors such as psychogenic polydipsia is unknown. Of note, some bipolar patients may have psychogenic polydipsia in which the polydipsia is primary and the polyuria is secondary whereas with lithium, the polyuria is primary.

### Risk of end stage renal Disease (ESRD)

Even though lithium substantially increases the risk of CKD Stage 3, if the renal insufficiency stays static and does not progress further, the risk/benefit ratio would overwhelmingly favor the continued use of lithium. Thus, the most important question in exploring the relationship between lithium and renal function is whether lithium can cause severe renal disease (typically defined as CKD stage 4–5) and/or true end stage renal disease (ESRD), typically defined as an eGFR < 15 ml/min and leading to either renal dialysis or transplantation. A number of studies, cross-sectional as well as longitudinal studies (Aiff et al., 2014a; Aiff et al., 2014b; Aiff et al. 2015; Bendz et al. 2010; Close et al. 2014: Rej et al., 2014b; Roxanas et al. 2014), supplemented by small case series (Gitlin 1993, 2023: Azab et al. 2015) have examined this issue.

The most persuasive data are those from the Swedish cohort of patients on either dialysis or renal transplantation in two geographical areas, totaling 2.8 million inhabitants (Aiff et al., 2014a; Aiff et al., 2014b; Aiff et al. 2015). ESRD was more prevalent among lithium users than non-lithium users with a risk ratio of 7.8 (Aiff et al., 2014a). Consistent with other earlier studies, the lithium/ESRD patients had been treated for a very long time with a mean time of 27 years (12–39). A follow-up study, however, suggested that the lower lithium levels used in more recent cohorts may substantially reduces this risk (Aiff et al., 2014b). In a follow-up of patients who started lithium during the observation period, 5% had progressed to CKD 4–5 (Aiff et al. 2015). As acknowledged by the authors (Aiff et al., 2014a; Aiff et al., 2014b; Bendz et al. 2010), patients in these studies may be misclassified, either because they did not disclose or forgot about their lithium treatment or escaped registration in the Swedish Renal Registry, thereby making their findings less definitive. Additionally, although many of these studies (Aiff et al., 2014a; Aiff et al., 2014b; Bendz et al. 2010) attempted to examine potential factors for ESRD other than lithium in their study population, it must be acknowledged that other risk factors such as other medications, the effect of psychiatric disorders or some medical comorbidities might still explain some of the findings.

A few case series have highlighted the vulnerability of short term follow-up studies in missing what are long term clinical complications (Azab et al. 2015; Gitlin 2023). Although the nature of these case series preclude estimating reliable probabilities of ESRD from lithium, they demonstrate that some lithium treated patients, regardless of whether they continue lithium may, even decades later, show progressive renal insufficiency, potentially leading to ESRD, dialysis or renal transplantation.

Finally, one recent study demonstrated, not surprisingly, that individuals with higher serum creatinine before beginning lithium treatment were far more likely to progress to CKD 4–5 vs. those with normal serum creatinine level pre-lithium (48% vs. 10%, p < .001, with a hazard ratio of 6.7) (Golic et al. 2021).

## Course of renal function after lithium discontinuation due to CKD

The trajectory of renal function after lithium discontinuation has been reported to stabilize or improve in some studies (Pahwa et al. 2021; Kessing et al. 2017; Bocchetta et al., 2015) while others found a subgroup of patients whose renal function continued to deteriorate after lithium discontinuation (Aiff et al., 2014a; Presne et al., 2003; Lepkifker et al. 2004).

In the most recent study, 13 patients whose eGFR at the time of lithium discontinuation was < 60 ml/minute, were followed for a median duration of 5.9 years (Hoekstra et al. 2022). The six (of thirteen) patients (46%) who showed a continued decrease in their eGFR after the lithium discontinuation had a lower mean eGFR (32 ml/min) compared to the seven patients whose eGFR increased after lithium discontinuation (eGFR = 46 ml/min) despite similar average duration of prior lithium exposure. This is consistent with earlier studies demonstrating that after a certain amount of renal damage, renal function will then continue to deteriorate in the absence of toxic factors (Presne et al., 2003; Markowitz et al. 2000). The earlier studies also suggested that the eGFR at the time of lithium discontinuation may be the best predictor of the trajectory of renal function after lithium discontinuation. Markowitz et al. (2000) found that serum creatinine values >2.5 mg/dl predicted further deterioration of renal function, while Presne et al. (2003) suggested a cutpoint of creatinine clearance of 40 ml/minute for progressive renal deterioration. Both of these results are consistent with those seen in Hoekstra et al. (2022). Continued deterioration of renal function after lithium discontinuation may also reflect other comorbid disorders that affect renal function such as hypertension, diabetes mellitus and/or nicotine dependency.

## Clinical implications and recommendations

Given the information described above, how should clinicians navigate the lithium/renal function issue? A number of recommendations seem clear while others are less certain. Therefore, these recommendations reflect our best judgment at the moment, acknowledging a lack of definitive data in a number of areas.

1. Evaluating renal function before lithium treatment:
  - Unquestionably, baseline renal function, as measured by eGFR, is a mandatory component of a pre-lithium workup. Serum creatinine levels, which were the most common measurement of renal function in the past are less useful since they are affected by muscle mass, which differs both across individuals and with age. (Older individuals have lower muscle mass and thus, may have lower serum creatinine for that reason alone). eGFR, which is now available in most clinical laboratories, is not dependent on muscle mass and is therefore a more reliable indicator of renal function. No other test of renal function-e.g., urine osmolality, 24 h urine measurement- should be a standard component of a pre-lithium workup.
2. Lithium regimen:
  - There is convincing data that low trough lithium levels are protective of renal tubular function. Therefore, once daily lithium, preferably using immediate release preparations (typically capsules), will minimize polyuria and preserve renal tubular function. However, this may only be true for the first five years of lithium treatment- i.e., before the functional renal tubular impairment becomes structural. Therefore, in the first few years of treatment, the goal of once daily lithium capsules is paramount. For patients who have been on lithium more chronically, the regimen-once daily vs. twice daily, immediate release vs. sustained release preparations-matters less.
3. Evaluation and treatment of lithium-induced polyuria:
  - Regular monitoring of polyuria by 24 h urine collections to evaluate whether the patient meets criteria for nephrogenic diabetes insipidus (3,000 ml/day) should not be routine. Decisions about whether to treat lithium-induced polyuria should be dictated by how distressing and disruptive these symptoms are for an individual patient, not the actual 24 h urine volume.
  - If lithium-induced polyuria is moderately distressing or if nocturia is substantially disruptive of sleep, treatment with amiloride or hydrochlorthiazide is indicated. Lithium treated patients who are benefitting from it should not discontinue lithium due to polyuria until these treatments have been considered and tried.
4. Management of patients during lithium treatment in order to minimize renal effects:
  - Table 2 summarizes the recommendations for minimizing lithium induced renal effects.
  - eGFR should be measured regularly. Although we (MG and MB) obtain these measurements every six months to yearly, others recommend every three month screening for renal function.
  - Substantial progression of renal deterioration can be defined as a decline in eGFR > 5 ml/minute within one year or as > 10 ml/minute within five years (Crowe et al. 2008). A patient whose eGFR decreases substantially or falls below 60 ml/minute should be shifted from a screening paradigm to a monitoring schedule. In this situation, eGFR should be monitored no less than every six months and possibly every three months.
  - A reasonable but not definitive paradigm would be that referral to a nephrologist for further evaluation of renal function should be considered for a lithium treated patient if/when the eGFR falls below 60 ml/minute or if the patient shows substantial deterioration of renal function as defined above. The goal of the consultation would be to explore other possible etiologies of renal insufficiency other than lithium. These would include inadequately treated diabetes mellitus, hypertension, polycystic renal disease, smoking, and so forth. The exception to this recommendation might be in older individuals (e.g., 60 years or older) for whom the normative eGFR values are lower. As an example, more than 1/3 of patients aged 70 or older have an eGFR < 60 ml/minute (Coresh et al. 2007).
  - In consultation with the nephrologist, the treating psychiatrist should have a discussion with the patient regarding the risks vs. benefits of staying on lithium if the eGFR is < 45–50 ml/minute or lower. Some patients for whom lithium has provided substantial or profound benefits or if the patient is older, might consider staying on lithium and having renal function even more closely monitored. Others may elect to discontinue lithium. If the decision is made to discontinue lithium, given the very slow progression-over years, not weeks to months- the new mood stabilizer should be added, titrated to optimal dose and then, the lithium should be slowly tapered. Sudden discontinuation of lithium in the face of lithium induced renal dysfunction is rarely if ever indicated.

**Table 2:** Clinical management of lithium treatment to minimize the risk of chronic kidney disease

- Regular monitoring of renal function, as measured by eGFR, every three months to yearly for most patients
- Increase frequency of measurements in patients who show a decrease in eGFR
- Optimally, 12 h lithium levels 0.6–0.8 mEq/L (see Nolen et al., 2019)
- Once daily lithium administration (if in the first five years of lithium treatment)
- Avoid lithium toxicity episodes (e.g. by regular patient and caregiver education including the risk constellations for intoxication and how to avoid it)
- Aggressive treatment of other comorbid disorders potentially affecting renal function such as diabetes mellitus and hypertension

## Conclusion

Treatment with long term lithium therapy confers a risk of renal dysfunction. This is commonly manifested by polyuria and thirst, reflecting renal tubular dysfunction which is initially functional but evolves over years into structural changes. A smaller percentage of lithium treated patients will show a decrease in glomerular filtration function as measured by eGFR. A very small percentage of lithium treated patients will progress to ESRD. However, appropriate monitoring of renal function before and during lithium treatment, keeping lithium levels in the moderate range, avoiding lithium intoxication (risk of kidney damage) and aggressively managing comorbid medical disorders, smoking cessation and avoiding potentially kidney damaging drugs likely to also affect renal function may decrease this risk.

## Data Availability

Not applicable.
